# Examining horizontal inequity and social determinants of inequality in facility delivery services in three South Asian countries

**DOI:** 10.7189/jogh.08.010416

**Published:** 2018-06

**Authors:** Tanvir M Huda, Alison Hayes, Michael J Dibley

**Affiliations:** 1 Sydney School of Public Health, University of Sydney, Sydney, Australia; 2 Maternal and Child Health Division, International Centre for Diarrhoeal Disease Research, Bangladesh

## Abstract

**Background:**

The utilization of maternal health care services has increased in many developing countries, but persistent wealth-related inequalities in use of maternal services remained an important public health issue. The paper examined the horizontal inequities and identified the key social determinants that can potentially explain such wealth-related inequalities in use of facility delivery services.

**Methods:**

The countries studied are Bangladesh, Pakistan and Nepal. We used horizontal inequity index to measure the horizontal inequity and decomposition of concentration index method to assess the contribution of different social determinants towards the wealth-related inequality. We have used household and women data from demographic and health surveys of Bangladesh (BDHS 2014), Pakistan (PDHS 2012-13) and Nepal (NDHS 2010-11).

**Results:**

All three countries showed pro-rich inequality in use of facility delivery services (Observed Concentration Index: Bangladesh = 0.235; Pakistan = 0.141; Nepal = 0.263). The study showed if the utilization were solely based on need factors there would have been little disparity between the rich and the poor (Need expected Concentration Index: Bangladesh = 0.004; Pakistan = 0.004; Nepal = 0.008). The use of facility delivery remained pro-rich in all three countries after taking the need factors into account (Horizontal inequity Index: Bangladesh = 0.231; Pakistan = 0.137; Nepal = 0.254). The decomposition analysis revealed that facility delivery is driven mostly by the social determinants of health rather than the individual health risk. Household socioeconomic condition, parental education, place of residence and parity emerged as the most important factors.

**Conclusions:**

Our study reiterates the importance of addressing social determinants of health in tackling wealth-related inequalities in use of facility delivery services. Health policy makers should acknowledge the importance of social determinants in determining individual health-seeking behaviour and accordingly set their strategies to improve access to facility delivery.

Globally, the maternal mortality rate (MMR) has fallen by almost 44% since 1990 from 385 maternal deaths per 100 000 live births to 216 maternal deaths per 100 000 live births in 2015 [1]. Around 800 women die each day worldwide due to maternal causes, 99% of these are in low or middle-income countries [1].

Maternal health thus remains a priority for the post-2015 Sustainable Development Goals (SDGs) [2]. Unlike the Millennium Development Goals (MDGs), where there was insufficient attention on equity, SDGs has a strong focus on equity. SDG 3 calls for ensuring healthy lives and promoting well-being for all, while SDG 10 calls for reducing inequality within and between countries to promote the inclusion and empowerment of all [3]. Countries are committed to achieving the SDGs without leaving behind anyone. For maternal health care, this means all countries need to continue building momentum in reducing maternal deaths but with a greater focus on reducing inequalities between different population groups. It is worth mentioning that some health inequalities are attributable to biological variations or free choice and unavoidable while others are due to uneven distribution of social determinants of health and are avoidable. Such avoidable inequalities in health between groups of people within and between countries are termed as health inequity [4].

The large inequalities in the risk of maternal death are mainly attributable to differences in utilization of maternal health services [5]. Obstetric complications account for 73% of global maternal deaths [6]. Hence, universal access to skilled birth attendants and emergency obstetric care are considered as the key interventions in preventing maternal deaths [7,8]. Expanding the coverage of these key maternal interventions must be accompanied by the reduction in widespread wealth-related inequality that exists within countries.

Despite a significant increase in the utilization of facility delivery services, wealth-related inequality in the use of facility delivery services remains a substantial problem in many low- and middle-income countries [9]. Studies have shown that wealth-related inequalities in the use of facility delivery services are much greater than wealth-related inequalities in the use of other priority health care services [10-14]. However, there could typically be inequality in the utilization of any health care. Sometimes differences in health care need can be legitimately attributable to differences in health service utilization. Also in low-income countries, poor usually utilize less health care services despite their greater need because of lack of purchasing power and high out of pocket expenses.

Since variation in use of facility delivery services could be due to differences in health care need (ie, women with complications may use facility delivery services more than women with no complications), wealth-related inequality itself could not be considered inequity. So it is important that we assess the extent to which use of facility delivery services is related to household economic status after differences in need across different wealth quintiles are accounted for. This concept is known as horizontal inequity, a widely accepted concept in health inequality research [15-18]. Horizontal inequity in health service utilization assesses the degree to which health care utilization is related to household economic condition after adjusting for differences in need across the different populations [17,19].

According to the World Health Organization around 22% of global maternal deaths in 2015 occurred in South Asia, which is home to 1.6 billion people [20]. The countries in this region also reported low to moderate levels of coverage in facility delivery services. There are several studies that have measured wealth-related or income inequalities in the use of key maternal heath interventions in South Asia region. However, very few have measured the wealth-related or income inequalities in use of facility delivery services after controlling for need factors. Also, there is no study that has measured the extent and identified sources of wealth-related inequalities in use of facility delivery services across South Asian countries.

The objective of this study is to examine the horizontal inequity in access to facility delivery in Nepal, Pakistan and Bangladesh, and identify the different need factors as well as other social determinants that can potentially explain such inequity in the use of the facility delivery services in these countries. It is essential to assess the horizontal inequity as well as social determinants that provide the greatest contribution towards the wealth-related inequality for informed policy decision making for reducing such inequalities in future.

## METHODOLOGY

### C**ountry settings**

About 24% of global maternal deaths occurred in South Asia. India, Pakistan, Bangladesh and Nepal contributed the most to the total number of maternal deaths in South Asia [20]. We have included Bangladesh, Nepal and Pakistan in this study, because all have had a recent demographic and health survey (2010 or later). India was excluded since no survey data after 2010 was available at the time of this study.

The inclusion of these countries provides a comparable landscape for examining horizontal inequity in the utilization of facility delivery services. The countries included in the study had a similar level of achievements in terms of MDG5. Nepal achieved 72% reduction in MMR from 1990 to 2015 and thus reached their MDG 5 [20]. Bangladesh also made a significant reduction in maternal mortality with an annual rate of reductions of 4.7%, one of the fastest rates of reduction among low-income countries [20]. However, Pakistan had relatively slower progress with an annual reduction of MMR of 3.5% [1]. According to the most recent Demographic Health Surveys, in Nepal, 35% of births took place in a health facility, while in Bangladesh 37%, and in Pakistan 48% [21-23].

### Data source

Demographic health surveys are nationally representative household surveys conducted approximately every 5 years in many low- and middle-income countries. We selected the most recent survey available for each country: Bangladesh (2014), Pakistan (2012-13) and Nepal (2010-11). The data for analysis was obtained from the household and women’s questionnaires. The latter was administered to all women age 15–49 years who spent the night before the survey in each household. The household questionnaire recorded demographic information on household members and socioeconomic conditions, while the women questionnaire recorded complete birth histories, including information on the use of maternal and child health services. In the Bangladesh DHS, information regarding maternal health care including place of delivery was only available for women who had given birth in the three years preceding the survey. To make the data comparable we restricted our entire analysis to women who had given birth in the three years preceding the survey.

### Outcome and economic indicator variables

Our outcome variable is whether or not a woman had facility delivery for their most recent live birth (Yes/No). Facility delivery was defined as a birth, attended at a either private or public health care facility. The economic indicator used was household wealth index score as a proxy of socioeconomic status since DHS does not collect information on household income or expenditure. The wealth index score was based on household ownership of consumer goods, household characteristics, drinking water source, toilet facilities and other characteristics related to the household’s socio-economic status. The asset indices were constructed using principal component analysis (PCA) [24].

### Measurement of horizontal inequity

We used horizontal inequity index developed by Van Doorslaer, Wagstaff, and others to measure the horizontal inequity in use of facility delivery services ([15-19,25].

### Need factors

The first step in developing horizontal inequity index is to define health care needs. We considered any complication identified during prenatal visits (Yes/No), and history of miscarriage, stillbirth or abortion (Yes/No) as the “need” factors. Complications during pregnancy can pose a serious threat to the mother and child and constitute a greater need for a skilled birth attendant, or delivery at a designated health care facility [26]. Also a previous adverse obstetric history is a risk factor for maternal and neonatal mortality [27].

### Non-need factors

We also identified the “non-need” factors that could potentially influence the facility-based delivery and are available in our data set. These were: women's age during the birth (<20 years, 21 to 34 years, 35 to 49 years); women’s decision taking power regarding her own health; region; area of residence (Urban and Rural); women’s education (no education/ primary/ secondary/ tertiary); husband’s education (no education/ primary/ secondary/ tertiary); wealth index; current employment status of the women and parity.

### Standardization facility delivery services use and measurement of horizontal inequity index

We can write the relationship between observed utilization, need and non-need factors using the following equation:
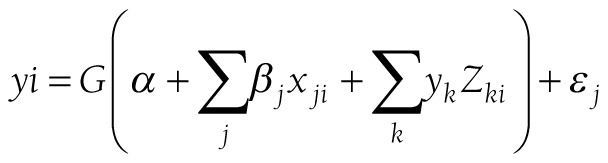


where y is the observed utilization of facility delivery services in binary form for any individual i, x_j_ is a vector of need variables and Z_k_ is a vector of non -need variables. G takes particular form for a probit model. If there are no non-need variables in the equation, then observed utilization of facility delivery services obtained from the equation could be interpreted as need-expected utilization.

The next step is to standardize utilization of facility delivery services for differences in need. We used indirect standardization method, which gives the difference between the actual distribution of facility utilization and the distribution that would be expected, given the distribution of need (ie, need expected distribution). The need-standardized health care utilization can be presented using the following equation:


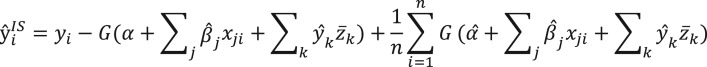


i.e., Need-standardized utilization = observed utilization – need-expected utilization.

Need-standardized utilization is the level of utilization that is observed among different socio economic groups after controlling for the need factors. Any difference in the need-standardized utilization can be attributable to non-need factors (eg, household socioeconomic condition, education).

We then calculated horizontal inequity index as the difference between the concentration index of observed utilization and the concentration index of need-expected utilization [15,19,28]:


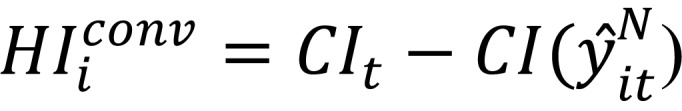


(Horizontal inequity index = CI of observed utilization − CI of need-expected utilization)

The horizontal inequity index denotes the degree of inequalities that exists across different socioeconomic groups after controlling for the need. A zero horizontal inequity index will indicate utilization of facility delivery services are according to need and proportionately distributed across different socioeconomic group [15]. A positive horizontal inequity index will indicate, there is pro-rich inequality in utilization of facility delivery services after controlling for need factors. Such inequalities can be attributable to non-need factors (eg, household socioeconomic condition, education). The details of the methods have been published elsewhere [15].

### Decomposition of socioeconomic inequalities in facility delivery services utilization

Finally, we decomposed the concentration index to obtain the contribution of both need factors and non-need social determinants to overall wealth-related inequality in utilization of facility delivery services using methods suggested by Wagstaff and others [15,16,19]. The contribution of each individual factor to the overall wealth-related inequality depends on two things. First its impact on the use of facility delivery services (elasticity) and second the degree of unequal distribution across different socioeconomic groups (concentration index). A factor that has high impact but little variation across different wealth quintiles will contribute minimally to the overall inequality. All analyses were carried out using Stata Version 13 (StataCorp, College Station TX, USA).

### Ethical consideration

All procedures and questionnaires used in BDHS 2014, PDHS 2012-13 and NDHS 2010-11 surveys were reviewed and approved by ICF Institutional Review Board (IRB) and by an IRB in the host country. All survey respondents gave informed consent before participation and all information was collected confidentially. All data are publicly available with all identifier information removed from the DHS Program (http://dhsprogram.com/data/available-datasets.cfm) for researchers who submit a request and meet the criteria for access to the data.

## RESULTS

**Table 1** shows the observed, need-expected and need-standardized utilization of health facility for delivery care within three years prior to the survey. We have reported these utilization rates by wealth quintiles. In all three countries, the observed utilization was lowest among the poorest and highest among the richest. Compared to Pakistan, the difference in coverage was more pronounced in Nepal and Bangladesh. The need-expected utilization of facility-based delivery was similar across the socioeconomic groups. We find large differences between observed and need expected utilization in all three-study countries. In the lowest quintile, the difference ranges from -25% to -29% indicating under-utilization of services while in the highest quintile, the difference ranges from 19% to 33% indicating over utilization of services given our definition of need. We then find substantial disparities in need-standardized utilization, which indicates the presence of horizontal inequities in utilization of facility delivery services across wealth quintiles.

**Table 1:** Distribution of observed, need-expected and need-standardized facility delivery utilization rates across different wealth quintile, Bangladesh (2014), Pakistan (2012-13) and Nepal (2012-2013)

	Poorest	Second	Middle	Fourth	Richest
**Bangladesh:**					
Observed*	22.56%	34.39%	47.13%	58.27%	77.99%
Need expected†	47.91%	48.05%	48.42%	48.42%	48.82%
Observed minus need expected	-25.35%	-13.66%	-1.30%	9.86%	29.17%
Need standardized‡	22.97%	34.66%	47.03%	58.18%	77.49%
**Pakistan**:					
Observed	44.84%	55.50%	63.61%	78.65%	89.79%
Need expected	69.64%	70.09%	70.32%	70.73%	70.85%
Observed minus need expected	-24.80%	-14.58%	-6.70%	7.92%	18.93%
Need standardized	45.52%	55.74%	63.62%	78.25%	89.26%
**Nepal:**					
Observed	21.41%	34.05%	45.18%	60.60%	85.22%
Need expected	50.08%	49.83%	49.66%	50.83%	52.03%
Observed minus need expected	-28.67%	-15.78%	-4.48%	9.77%	33.20%
Need standardized	21.82%	34.71%	46.01%	60.26%	83.68%

*Observed or unstandardized CI: The inequality that is observed among different socio economic groups. Any inequality in observed utilization is attributed to both need and non-need factors.

†Need-expected CI: The inequality in utilization of health services that would be expected, given the distribution of need

‡Need-standardized CI: The inequality that is observed among different socio economic groups after controlling for the need factors. Any inequality in need-standardized utilization is attributed to non-need factors only.

**Figure 1:**
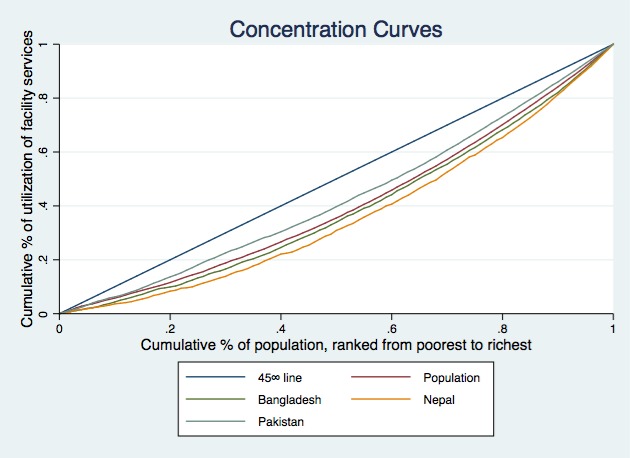
Concentration curves of observed socioeconomic-related inequality in facility delivery services in Bangladesh, Nepal and Pakistan.

**Figure 2:**
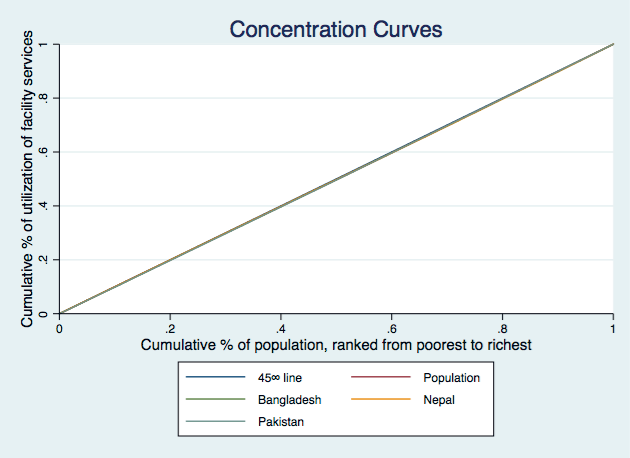
Concentration curves of need predicted socioeconomic-related inequality in facility delivery services in Bangladesh, Nepal and Pakistan.

**Figure 3:**
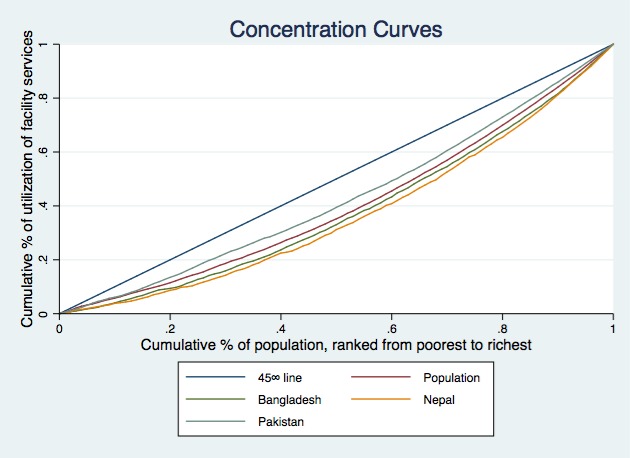
Concentration curves of standardized socioeconomic-related inequality in facility delivery services in Bangladesh, Nepal and Pakistan.

**Figure 1****, ****Figure 2** and **Figure 3** present the concentration curves for observed, need-expected and need-standardized utilization for all three countries. In **Figure 1**, the concentration curves for observed utilization of facility delivery services for all three countries lie below the line of equality, which indicates significant pro-rich inequalities. In **Figure 2**, the concentration curves of need-expected utilization lie very close to the line of equality, which means there is very little inequality in need-expected utilization. In **Figure 3**, the concentration curves for need-standardized distribution lie below the line of equality. This suggests there is considerable pro-rich inequality in use of facility delivery services after taking account of need factors.

The concentration index (CI) values for the corresponding concentration curves have been presented in **Table 2****.** The results suggest that among three countries, Nepal has the most pro-rich inequality in use of facility delivery services (CI = 0.263). Bangladesh has slightly lower inequality with a positive concentration index of 0.235 while Pakistan has the least inequality (CI = 0.141) among the three countries.

**Table 2:** Concentration index of observed, need-expected and need-standardized facility delivery utilization, Bangladesh (2014), Pakistan (2012-13) and Nepal (2012-13)

	CI	SE	95% CI		*P*-value	t ratio
Bangladesh:						
Observed or unstandardized*	0.235	0.010	0.216	0.253	0.000	24.611
Need expected†	0.004	0.001	0.002	0.005	0.000	5.097
Need standardized‡	0.231	0.010	0.212	0.250	0.000	24.270
Pakistan:						
Observed or unstandardized	0.141	0.006	0.129	0.153	0.000	22.652
Need expected	0.004	0.001	0.003	0.005	0.000	5.887
Need standardized	0.137	0.006	0.125	0.149	0.000	22.236
Nepal:						
Observed or unstandardized	0.263	0.011	0.241	0.285	0.000	23.526
Need expected	0.008	0.002	0.005	0.012	0.000	5.064
Need standardized	0.254	0.011	0.233	0.276	0.000	23.052

CI – concentration index, 95% CI – 95 confidence interval, SE – standard error

*Observed or unstandardized CI: The inequality that is observed among different socio economic groups. Any inequality in observed utilization is attributed to both need and non-need factors

†Need-expected CI: The inequality in utilization of health services that would be expected, given the distribution of need.

‡Need-standardized CI: The inequality that is observed among different socio economic groups after controlling for the need factors. Any inequality in need-standardized utilization is attributed to non-need factors only.

The concentration indices for need-expected utilization were close to zero for all three countries (Bangladesh 0.004, Pakistan 0.004, Nepal 0.008). Which means if the use of facility delivery services were solely based on need factors, there would have been no inequality among the different wealth quintiles. Finally, the results for need-standardized concentration index (horizontal inequity Index) show significant pro-rich inequities in all the three countries. The horizontal inequity indices were close to the concentration index for observed utilization for all three countries (Bangladesh 0.231, Pakistan 0.137 and Nepal 0.254).

In order to illustrate which social determinant of health have contributed to horizontal inequity, we presented the decompositions of the concentration index into its components **(****Table 3****).** We find most of the results were consistent across countries. The results demonstrate that the combined contribution of all “need” factors (as reported in the sub-total row) was very small in all the countries (Bangladesh 1.6%, Pakistan 2.2% and Nepal 3.5%). Among the “non-need” social determinants, wealth had the greatest contributions towards horizontal inequality in utilization of facility delivery services (Bangladesh 62.9%, Pakistan 55.1% and Nepal 57.1%).

**Table 3:** Decomposition analysis of socioeconomic-related inequality in facility delivery Bangladesh (2014), Pakistan (2012-13) and Nepal (2012-13)

	Bangladesh				Pakistan				Nepal			
	**Elasticity**	**CI**	**Abs. Cont.**	**Rel. Cont.**	**Elasticity**	**CI**	**Abs. Cont.**	**Rel. Cont.**	**Elasticity**	**CI**	**Abs. Cont.**	**Rel. Cont.**
**Any complication:**												
No												
Yes	0.069	0.052	0.004	1.55%	0.069	0.070	0.005	3.42%	0.269	0.030	0.008	3.07%
**Previous history of pregnancy termination:**												
No												
Yes	0.008	0.024	0.000	0.08%	0.027	-0.030	-0.001	-0.57%	0.025	0.021	0.001	0.20%
Subtotal				1.6%				2.2%				3.5%
**Mother’s age at birth (years):**												
<20												
20-34	0.132	0.036	0.005	2.00%	0.044	0.033	0.001	1.02%	-0.063	0.035	-0.002	-0.84%
34-49	0.013	-0.047	-0.001	-0.27%	0.010	-0.115	-0.001	-0.85%	0.000	-0.304	0.000	0.03%
Parity												
0-1												
2	0.158	0.061	0.010	4.08%	0.053	0.109	0.006	4.13%	0.179	0.135	0.024	9.19%
-10	0.034	0.037	0.001	0.54%	0.026	0.104	0.003	1.91%	0.032	0.083	0.003	1.02%
**Can make decision about her own health:**												
No												
Yes	0.018	0.037	0.001	0.28%	0.014	0.054	0.001	0.55%	0.071	0.043	0.003	1.15%
**Husband’s education level:**												
No education												
Primary	0.036	0.003	0.000	0.05%	0.010	-0.091	-0.001	-0.68%	0.015	-0.039	-0.001	-0.21%
Secondary	0.012	0.297	0.004	1.51%	0.003	0.105	0.000	0.22%	0.011	0.296	0.003	1.28%
Tertiary	0.057	0.457	0.026	11.03%	0.026	0.403	0.011	7.54%	0.017	0.474	0.008	3.00%
**Women’s education level:**												
No education												
Primary	0.052	-0.029	-0.001	-0.64%	0.014	0.124	0.002	1.26%	0.040	0.141	0.006	2.14%
Secondary	0.021	0.306	0.007	2.77%	0.019	0.352	0.007	4.79%	0.033	0.443	0.015	5.64%
Tertiary	0.053	0.494	0.026	11.19%	0.041	0.566	0.023	16.37%	0.035	0.646	0.022	8.52%
**Region:**												
Barisal/Punjab/Mountain												
Chittagong/Sindh/Hill	0.000	0.160	0.000	-0.02%	0.048	-0.039	-0.002	-1.32%	0.066	-0.108	-0.007	-2.73%
Dhaka/Khyber pakhtunkhwa/Terai	0.022	0.183	0.004	1.72%	-0.008	0.001	0.000	-0.01%	0.096	0.231	0.022	8.48%
Khulna/Balochistan	0.063	-0.036	-0.002	-0.96%	-0.021	-0.126	0.003	1.92%				
Rajshahi/Gilgit	0.041	-0.055	-0.002	-0.97%	0.018	-0.411	-0.007	-5.28%				
Rangpur/Islamabad	0.025	-0.250	-0.006	-2.65%	0.016	0.515	0.008	5.95%				
Sylhet	-0.005	-0.019	0.000	0.04%								
**Area of residence:**												
Urban												
Rural	-0.138	-0.191	0.026	11.27%	-0.009	-0.302	0.003	1.91%	-0.296	-0.145	0.043	16.33%
**Wealth:**												
Poorest												
Second	0.033	-0.525	-0.017	-7.32%	0.029	-0.519	-0.015	10.51%	0.056	-0.323	-0.018	-6.89%
Middle	0.071	-0.161	-0.011	-4.86%	0.039	-0.142	-0.005	-3.89%	0.082	0.066	0.005	2.06%
Fourth	0.120	0.272	0.033	13.89%	0.066	0.281	0.018	13.07%	0.092	0.452	0.042	15.87%
Richest	0.190	0.756	0.144	61.23%	0.106	0.751	0.080	56.50%	0.147	0.823	0.121	46.13%
**Women's current employment status:**												
Not working												
Working	-0.033	-0.109	0.004	1.55%	-0.009	-0.243	0.002	1.52%	-0.080	-0.165	0.013	5.05%

CI – concentration index, Abs. Cont – absolute contribution, Rel. Cont – relative contribution

Education of both women and their husband had a positive association with use of facility delivery services as indicated by their positive elasticity, and were also concentrated among the wealthy participants. Hence, we find a very high contribution of education towards horizontal inequality. The overall contribution of women’s education towards horizontal inequality was 13.3% for Bangladesh, 22.4% for Pakistan and 16.3% for Nepal. The contribution of husband’s education was more pronounced for Bangladesh (12.6%) but much lower for Pakistan (7.0%) and Nepal (4.0%).

The place of residence was also found to be an important factor for wealth-related inequality for both Bangladesh (11.3%) and Nepal (16.3%). Parity had a much greater contribution to horizontal inequality in Nepal than Bangladesh and Pakistan. Similarly, women’s employment status also had a much greater contribution to horizontal inequality in Nepal than the other two countries. The decision-making ability had a very minimal contribution in horizontal inequality in all three countries. Women’s age at the time of birth also had minimal contribution to horizontal inequality in Bangladesh while no contribution in other two countries.

## DISCUSSION

Our study results showed large horizontal inequities in the use of facility-based delivery services favoring the better off than the poorer individuals in all three-study countries. We found very minimal differences between observed inequality and horizontal inequity. This was because ‘need’, as defined in our study (any complication during pregnancy and poor obstetric history) were equally distributed among the all-socioeconomic groups. Hence the distributions of need expected utilization were similar across different socioeconomic groups.

Our results indicate that utilization of facility-based delivery services is much higher than needed for the rich, and much lower for the poor suggesting their greater unmet need for such care. Since the SDGs call for universal coverage of key maternal health services, overutilization of facility delivery services among the rich is not undesirable. Although in many cases it is leading to excess rates of caesarian section driving up the overall health care expenditure with some negative health outcomes. However, the low utilization of delivery services among the poor is a matter of great concern.

Lower rates of facility delivery services among the poor result from several factors as observed in the decomposition analysis. Inequalities in household wealth, maternal and paternal education, the area of residence and parity were found to be the main drivers of horizontal inequality in use of facility-based delivery care. The study findings reemphasize the role of social determinants in horizontal inequality in facility delivery services utilization.

Our study shows that the inequality in household wealth as one of the major contributors of horizontal inequity in access to facility delivery services. A study in Namibia also reported similar findings and concluded inequalities in wealth distribution as one of the main drivers of inequities in the distribution of skilled birth attendance [29]. Other studies have shown household wealth to be positively related to facility delivery care [29-36]. The underutilization of facility delivery services by the poor could be the result of high out of pocket payments associated with the cost of facility delivery including the cost of transportation, physician and cost of medications in both private and public health care facilities. A number of studies have reported significant out of pocket expenditure for maternal health services and socioeconomic inequalities in our study countries [37-39]. It is thus important to address this unacceptably high level of income inequality in these countries. One way would be to target the vulnerable populations and provide cash or in-kind incentives to promote facility delivery similar to Aama (Mothers’) Programme (cash transfer element) in Nepal; the Maternal Health Voucher Scheme in Bangladesh and the Sehat (Health) Voucher Scheme in Pakistan [40].

Education is a significant predictor of the place of delivery. Previous studies in Nepal, Bangladesh, Pakistan, and elsewhere, have shown educated women are more likely to use facility delivery services than those with limited or no education [35,36,41-47]. Studies have also shown that women with an educated husband use facility delivery services more than women with a less educated husband [31,36,43,46]. Health knowledge is deemed to be the underlying mechanism in the association between formal education and use of health services. Formal education increases understanding of health issues, which in turn leads to greater use of maternal and child health services [48]. An educated person will know the risks of home delivery. Thus education influences the use of health care services through improved knowledge, attitudes and practice [29].

Similar to a previous study in Ghana, Rwanda and the Philippines our results show both women’s and their husbands’ education were major drivers of horizontal inequity in the use of facility-based delivery services although the level of contribution differs between the countries (49). For female education, the contribution was more pronounced in Pakistan and Nepal than Bangladesh. Bangladesh has achieved a rapid expansion in education, especially female education. We see almost no inequalities in the distribution of secondary education across different socio economic populations in Bangladesh. The socioeconomic inequalities in tertiary education among the females are also less pronounced in Bangladesh than the other two countries. As a result, we see larger contribution of women’s education to socioeconomic inequalities in use of facility delivery in Nepal and Pakistan.

The other factor that made a similar contribution to the horizontal inequity across the three counties was residential location of the women. The area of residence accounted for almost one-sixth of the horizontal inequity in Nepal and one-tenth of the inequality in Bangladesh. There is a higher concentration of the economically worse off in rural areas in Nepal and Bangladesh compared to Pakistan. So although the area of residence showed strong association with the use of facility delivery services in Pakistan, its contribution towards horizontal inequity was not as pronounced as the other two countries. The inequality in use of facility delivery services across the area of residence can partly be explained by differential access to services favoring urban areas (ie, more availability of health care facilities for delivery care) and partly by the difference in the socioeconomic condition of the population residing in urban vs rural areas. Several other studies have shown the area of residence as an important determinant for the use of facility delivery services [29,33,36,42,46,50,51].

Parity also made a significant positive contribution to the horizontal inequity in use of facility delivery services in all three countries. Our findings are similar to the findings of a study conducted in Ghana, Rwanda and Philippines that showed parity as a significant factor for socioeconomic inequalities in the use of facility delivery services(49). Other studies in Nepal, Bangladesh and Pakistan have also found institutional delivery varied dramatically by parity [35,36,47]. It is possible that after a safe delivery at home some women would perceive subsequent deliveries of low risk or be less apprehensive about delivery and avoid facility delivery more than women who are pregnant for the first time [36,49]. The latter is of particular concern since all births are considered at risk and WHO recommends delivery by a skilled attendant for all births.

Need factors as defined by reported complications and poor obstetric history contributed minimally towards the use of facility delivery services. In our study, complications during pregnancy accounted for only 1.6-3.5% percent of the inequality. This was because the distribution of complications was equally distributed across different socioeconomic groups. Earlier studies have shown that complications during pregnancy are the important determinant of use of facility-based delivery services [31,36,41,44,51-53].

### Strengths and limitations

The major strength of our study is that we assessed horizontal inequity in use of facility delivery services using large nationally representative surveys for all three countries. Our major limitation is the scarcity of data regarding the health status of the mother that could have better assessed the need for higher-level delivery care. Another limitation is we did not include supply-side determinants including financial and physical access to health facilities, service readiness and service quality. We also used an asset-based wealth index as a proxy indicator of household economic status since there is no information on household income or expenditure. One of the major critiques of using household wealth index is that it fails to capture recent financial shock or loss of income for any reason.

## CONCLUSIONS

Our study findings demonstrate the importance of reducing inequalities in social determinants of health in order to increase the coverage of facility delivery services among the poorest women. Strategies to increase the number of health facilities alone may not be enough to ensure access to all population. People with the lower socioeconomic background will continue to use comparatively less facility delivery services. It is now well established that different social determinants of health produce the social hierarchy or social stratification that result in inequalities in access to health care services. Policy makers should thus try to tackle the underlying structural determinants of socioeconomic related health inequalities. But tackling social determinants of health is beyond the capacity of the ministry of health alone. A coordinated multi-sectoral approach will be needed to combat the wide spread socioeconomic related inequalities in use of facility delivery. In short term, programs may consider evidence based intervention including cash transfers to the mothers as well as performance-based incentives to the providers to increase access to facility delivery among the poor population.
